# Endorsement for improving the quality of reports on randomized controlled trials of traditional medicine journals in Korea: a systematic review

**DOI:** 10.1186/1745-6215-15-429

**Published:** 2014-11-05

**Authors:** Jiae Choi, Ji Hee Jun, Byoung Kab Kang, Kun Hyung Kim, Myeong Soo Lee

**Affiliations:** Medical Research Division, Korea Institute of Oriental Medicine, Daejeon, 305-811 South Korea; Division of Clinical Medicine, School of Korean Medicine, Pusan National University, Yangsan, South Korea

## Abstract

The aim of this study was to assess the endorsement of reporting guidelines in Korean traditional medicine (TM) journals by reviewing their instructions to authors. We examined the instructions to authors in all of the TM journals published in Korea to assess the appropriate use of reporting guidelines for research studies. The randomized controlled trials (RCTs) published after 2010 in journals that endorsed reporting guidelines were obtained. The reporting quality was assessed using the following guidelines: the 38-item Consolidated Standards of Reporting Trials (CONSORT) statement for non-pharmacological trials (NPT); the 17-item Standards for Reporting Interventions in Clinical Trials of Acupuncture (STRICTA) statement, instead of the 5-item CONSORT for acupuncture trials; and the 22-item CONSORT extensions for herbal medicine trials. The overall item score was calculated and expressed as a proportion.One journal that endorsed reporting guidelines was identified. Twenty-nine RCTs published in this journal after 2010 met the selection criteria. General editorial policies such as those of the International Committee of Medical Journal Editors (ICMJE) were endorsed by 15 journals. In each of the CONSORT-NPT articles, 21.6 to 56.8% of the items were reported, with an average of 11.3 items (29.7%) being reported. In the 24 RCTs (24/29, 82.8%) appraised using the STRICTA items, an average of 10.6 items (62.5%) were addressed, with a range of 41.2 to 100%. For the herbal intervention reporting, 17 items (77.27%) were reported. In the RCT studies before and after the endorsement of CONSORT and STRICTA guidelines by each journal, all of the STRICTA items had significant improvement, whereas the CONSORT-NPT items improved without statistical significance.The endorsement of reporting guidelines is limited in the TM journals in Korea. Authors should adhere to the reporting guidelines, and editorial departments should refer authors to the various reporting guidelines to improve the quality of their articles.

## Introduction

In cases in which full reporting information is inaccessible, billions of dollars are wasted, bias is introduced, and research and the care of patients are detrimentally affected [1]. Well-designed and well-conducted randomized controlled trials (RCTs) represent the best available methodology for evaluating the effects of health care interventions. In general, they deliver reliable results that could inform future research or clinical practice. Poorly executed trials with inadequate methodologies are associated with bias and might produce exaggerated effects of an intervention [2]. A thorough assessment of a trial's design, management and analysis is vital to assess the quality and reliability of its published research. This type of assessment is only possible if the trial report presents the critical information required for such an appraisal. High-quality reporting in evidence-based research articles is crucial for the dissemination and implementation of research findings, and reporting guidelines are useful tools for increasing the comprehensiveness, accuracy, and transparency of research studies [3]. The lack of clear, transparent, and sufficiently detailed reporting of RCTs is a barrier to an adequate appraisal of the quality and applicability of published trials [4].

In the mid-1990s, in response to concerns regarding the quality of reporting in RCTs, an international group of researchers, statisticians, epidemiologists and biomedical editors developed the Consolidated Standards of Reporting Trials (CONSORT) statement [5]. This statement has been endorsed by the World Association of Medical Editors, the International Committee of Medical Journal Editors (ICMJE) and the Council of Science Editors [6]. The CONSORT statement is a comprehensive guideline for reporting RCTs, and it is associated with trial design and implementation improvements [7]. Since the development of the CONSORT statement, several extensions and elaborations have been included to include reporting requirements for different types of trials and interventions such as herbal interventions [8], non-pharmacological treatments [9], reporting of harm [10], inferiority and equivalence trials [11], pragmatic trials and Standards for Reporting Interventions in Clinical Trials of Acupuncture (STRICTA) [12].

Almost 600 general and specialty journals endorse the CONSORT statement [13]. Although reporting guidelines play a central role in improving the quality of articles, there are considerable opportunities to improve the reporting of Korean clinical trials. There has been a considerable increase in the number of trials focused on traditional medicine (TM) and complementary and alternative medicine (CAM) in Korea [14]. The quality of Korean clinical medical research articles has consistently been a topic of discussion, and the guiding effect of TM journals should not be ignored; therefore, it is necessary to review the requirements outlined by Korean medical journals.

Considering these needs, several local studies assessing the endorsement of reporting guidelines by journals and authors are available [15–18]. Additionally, it is necessary to assess the TM journals in Korea that endorse reporting guidelines to determine whether the articles published in these journals provide satisfactory descriptions of the study design and intervention by adopting standards for all of the items in the reporting guides.

In this study, we aimed to investigate the extent to which Korean TM journals incorporated reporting guidelines into their instructions for authors. Any reference to the ICMJE was studied, and the quality of the reporting of RCTs in the TM journals that endorsed reporting guidelines was assessed.

## Review

### Methods

This study was reported in accordance with the guidelines from PRISMA (Preferred Reporting Items for Systematic Reviews and Meta-Analyses) [19].

### Selection of journals and RCTs

We included the TM journals from our previous article, which introduced all of the TM journals in a Korean medical database [20] and excluded those that did not have a website.

The instructions for the authors in each TM journal were downloaded, and the text referencing the CONSORT statement or other information relevant to the reporting guidelines for trials was examined. Additionally, we searched for any reference to the ICMJE’s Uniform Requirement for Manuscripts Submitted to Biomedical Journals. If the reporting guidelines were not referenced in the journals, we assumed that the journal had not adopted the reporting guidelines. We identified the RCTs in the journals that had adopted reporting guidelines by screening all of the issues. We retrieved the parallel group of RCTs included in the journals published after 2010 that had adopted reporting guidelines and assessed whether the use of reporting guidelines in the RCT reports was appropriate.

### Data extraction

One author (JC) reviewed the websites of the TM journals. The instructions for authors, manuscript submission documents and relevant information for authors were extracted as data sources, including guidelines or instructions pertaining to the domains of editorial policy. The data included the journal name, website, ISSN, general policy references and reporting guidelines.

The screening of the title, abstract and full text of potentially relevant RCTs was completed by two authors (JC and JHJ). The full-text reports of the RCTs published in journals that referred to reporting guidelines were downloaded. Two authors (JC and JHJ) independently reviewed these RCT reports to assess the quality of reporting and to determine whether flow diagrams were included and subsequently validated by the third reviewer (BKK). Disagreements were resolved by consensus or by the third and fourth authors of this study (KHK and MSL).

To ensure correct interpretation, the experienced systematic reviewers dedicated an extensive amount of time to discussing all of the reporting statement guidelines and independently assessing and scoring the reporting quality. The data were collected using a standardized form. To maximize accuracy, the data extraction was performed at least twice for each article during several months. The approach and assumptions for determining the study quality were discussed extensively with the other authors. Disagreements were resolved by discussion.

### Identification of reporting guidelines

The CONSORT group highly recommended that guideline users refer to the current version while writing or interpreting the reports of clinical trials [13]. In this study, the current version of the reporting guidelines was used to assess the quality of reporting in the TM journals that endorsed reporting guidelines. The extensions of the CONSORT statement were developed to provide additional guidance for RCTs with specific designs, data and interventions [13]. We attributed appropriate guideline tools for each TM intervention in the RCTs. The extension of the CONSORT statement for RCTs to non-pharmacological trials (NPT) [9] was based on the CONSORT guidelines, and for assessing an NPT the extension guidelines consider specific issues that might affect the treatment results (that is, surgery, technical interventions, rehabilitation, psychotherapy, behavioral interventions, implantable and non-implantable devices, and complementary medicine).

We selected the reporting guidelines as follows: the NPT extension of the CONSORT 2010 (38 items) [9], the CONSORT extension for herbal medicine RCTs (22 items) [8], and the 17-item STRICTA guideline that was designed to replace the 5 CONSORT items for acupuncture trials [12].

### Data analysis

Each item was rated using a dichotomous scale (that is, ‘reported’ or ‘not reported’). The rating of ‘reported’ was recorded in cases in which relevant information was at least partially reported in the article. The rating of ‘not reported’ was recorded when relevant information was completely missing in the article.

The extracted variables included the publication and reporting characteristics as well as the items rated as ‘reported’. The data were analyzed using Microsoft 2010 and SPSS WIN 12.0 K (SPSS, Inc., Chicago, IL, USA). To assess the adherence to the CONSORT-NPT guideline items, we calculated the number and proportion of articles describing each item. The sum of the scores was converted to a percentage value for the reported items of each article (the proportion of each item = the number of reported items/the total items) and each section (the proportion of each section = the percentage of the sum of items of each section/the total items of each section). Additionally, before-and-after comparisons were performed to investigate whether the reporting quality of the RCTs was altered after the journal endorsed the reporting guidelines. We presented the percentages and percentage differences with binominal 95% confidence intervals after the journal adopted the CONSORT-NPT and STRICTA reporting guidelines.

## Results

### Selection of the studies for analysis

**Figure 1:**
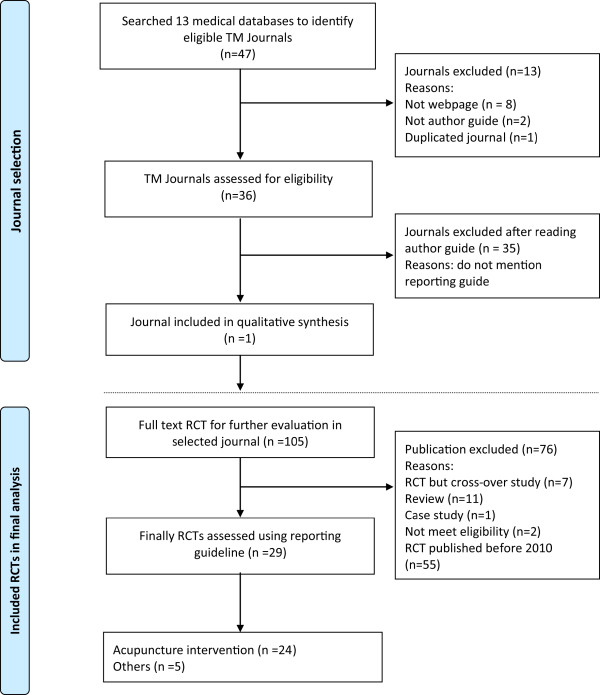
**Flowchart of journal and articles through the phase screening and eligibility evaluation.**

The selection process is presented in Figure 1. We identified 47 TM journals as potential candidates for our investigation of the adoption of reporting guidelines for clinical trials. Thirty-six journals were obtained from a Korean medical database. Of these journals, one journal recommends the use of reporting guidelines for clinical trials. To investigate the implementation of the reporting guidelines of the RCTs in this journal, the journal website was manually searched for all of the articles published from inception through December 2013.

After screening the abstracts and titles, we found 105 candidate articles. Subsequently, 76 articles were excluded because they were published before 2010 (55 trials). We additionally excluded the following: case studies (1), cross-over studies (7), reviews (11) and articles for other reasons (2). Lastly, 29 articles meeting our inclusion criteria were included, read completely and evaluated.

A total of 29 articles were eligible for the study. The types of interventions assessed in the 29 RCTs were as follows: acupuncture (24 trials), herbal medicine (1 trial), moxibustion (3 trials) and cupping (1 trial). The assessments were classified as non-pharmacological (96.6%) or pharmacological (3.4%).

### General editorial policy

General editorial policies such as those of the ICMJE were endorsed by 15 journals (15/36, 41.6%); however, the journals did not specifically refer to reporting guidelines in the context of the ICMJE endorsement. Of the 15 journals, 2 journals specifically mentioned particular policies such as the Helsinki Declaration on Ethical Principles for Medical Research Involving Human Subjects, the Committee on Publication Ethics (COPE), and the Institutional Animal Ethical Committee (AEC) NIH Guide for the Care and Use of Laboratory Animals (Table 1).

**Table 1:** **The list of traditional medicine (TM) journals searched in reporting quality review**

Journal title (Abbreviation)	ISSN	Website	Reference to general editorial policies ^a^	Reporting guidelines specific in instructions to authors ^b^
*The Journal of Korean Oriental Medicine* (J Korean Orient Med)	1010-0695	http://www.jkom.org/	ICNJE	nr
*Korean Journal of Acupuncture* (Korean J Acupunct)	2287-3368	http://www.kjacupuncture.org/	ICNJE	nr
*Korean Journal of Oriental Physiology & Pathology* (Korean J Orient Physiol & Pathol)	1738-7698	http://www.hantopic.com/kjopp/KJOPP.htm	nr	nr
*The Korea Journal of Herbology* (Korean J Herbology)	1229-1765	http://www.herbology.or.kr/	ICNJE	nr
*Journal of Oriental Sports Medicine* (J Orien Sports Med)	N/A	http://sskm.tistory.com/	nr	nr
*Journal of Korean Traditional Oncology* (J Korean Tradit Oncol)	1229-2621	http://www.koreanoncology.or.kr/	ICNJE	nr
*Journal of Pharmacopuncture* (Pharmacopuncture)	2093-6966	http://www.journal.ac/	nr	nr
*Journal of Acupuncture and Meridian Studies* (J Acupunct Meridian Stud)	2005-2901	http://journalams.com/00_Main.html	ICNJE	nr
*The Korean Journal of Oriental Preventive Medicine* (Korean J Orient Prev Med)	1226-7066	http://www.prehan.com/mainnew/main.html	ICNJE	nr
*The Journal of Korean of Medical GI-GONG Academy* (J Korean GG)	N/A	http://gigong.or.kr/index.php	nr	nr
*The Korean Journal of Joongpoong* (Korean J Joongpoong)	1598-1843	http://www.kmstroke.or.kr/v2	nr	nr
*Journal of Korean Acupuncture and Moxibustion Medicine Society* (J Korean Acupunct & Mox Med Sci)	1229-1137	http://www.acumoxa.or.kr/	nr	CONSORT, STARD, STROBE, QUOROM, MOOSE, STRICTA
*The Journal of Korean Oriental Internal Medicine* (J Korean Orient Intern Med)	1226-9174	http://www.oim.or.kr	nr	nr
*The Journal of Oriental Obstetrics and Gynecology* (J Orient Gynecol Obstet)	1229-4292	http://www.oobgy.or.kr/	nr	nr
*The Journal of Korean Oriental Pediatrics* (J Korean Orient Pediatr)	1226-8038	http://www.akop.or.kr/	ICNJE	nr
*Journal of Oriental Neuropsychiatry* (J Orient Neuropsychiatry)	1226-6396	http://www.onp.or.kr/	ICNJE	nr
*The Journal of Korean Oriental Medical Ophthalmology and Otolaryngology and Dermatology* (J Korean Orient Otolaryngol Dermatol)	1738-6640	http://ood.or.kr/	ICNJE	nr
*Journal of Oriental Medical Thermology* (J Orient Med Thermol)	1598-592X	http://www.komt.or.kr/	nr	nr
*The Journal of The Korea Institute of Oriental Medical Diagnostics* (J Korean Orient Med Diagn)	1226-5241	http://www.bmpomd.or.kr/	ICNJE	
*The Korean Journal of Oriental Medical Prescription* (Korean J Orient Med Prescription)	1229-1218	http://www.ompak.okdanche.com/	nr	nr
*The Journal of Korean Medical Classics* (J Korean Med Classic)	1229-8328	http://www.wonjeon.org/	nr	nr
*The Journal of The Society of HyungSang Medicine* (J Sci HyungSang Med)	1229-8328	http://www.hyungsang.or.kr/	nr	nr
*Journal of Sasang Constitutional Medicine* (J Sasang Constit Med)	1226-4075	http://www.esasang.com	nr	nr
*Journal of Somun Oriental Medical Society* (J Somun Orient Med)	1975-2407	http://www.somun.or.kr	nr	nr
*The Journal of Korea CHUNA Manual Medicine for Spine and Nerves* (J Korea CHUNA Med Spine & Nerves)	1598-1630	http://www.chuna.or.kr	ICNJE	
*The Journal of Korean Medical History* (J Korean Med Hist)	1229-6147	http://www.medicalhistory.or.kr/	nr	nr
*Journal of Society of Korean Medicine for Obesity Research* (J Sci Korean Med Obes Res)	1976-9334	http://www.obesity.or.kr/	ICNJE, Helsinki Declaration, NIH Guide for the Care and Use of Laboratory Animals	
*Journal of Oriental Rehabilitation Medicine* (J Orien Rehabil Med)	1229-1854	http://www.ormkorea.org/	ICNJE	nr
*Oriental Pharmacy and Experimental Medicine* (Orient Pharm Exp Med)	1598-2386	http://www.opem.org/	nr	nr
*Journal of East-West Nursing Research* (J East-West Nurses Res)	1226-4938	http://society.kisti.re.kr/~ewnri/	nr	nr
*Integrative Medicine Research* (Integr Med Res)	2213-4200	http://www.imr-journal.org/	ICNJE	
*Journal of Ginseng Research* (J Ginseng Res)	1226-8453	http://www.ginsengsociety.org/eng/	ICMJE, COPE, Good Publication Practice for Medical Journals Uniform Requirements for Manuscripts Submitted to Biomedical Journals: Writing and Editing for Biomedical Publication, Helsinki Declaration, AEC	
*Natural Product Sciences* (Nat Prod Res)	0253-3073	http://www.ksp.or.kr/	nr	nr
*Korea Journal of Pharmacognosy*	0253-3070	http://journal.ksp.or.kr/	nr	nr
*Korean Journal of Medicinal Crop Science* (Korean J Med Crop Sci)	0252-9777	http://www.medcrop.or.kr/	nr	nr
*International Journal of Genuine Traditional Medicine* (Int J Genuine Trad Med)	2233-8985	http://www.e-tang.org/journal.do?method=journalintro&journalSeq=J000027&menuId=&introMenuId=0101	nr	nr

AEC: Institutional Animal Ethical Committee; COPE: Committee on Publication Ethics; CONSORT: Consolidated Standards of Reporting Trials; ICMJE: International Committee of Medical Journal Editors; MOOSE: Meta-analysis of Observational Studies in Epidemiology; nr: not reported; QUOROM: Quality of Reporting of Meta-analyses; STARD: Standards for Reporting of Diagnostic Accuracy Studies; STROBE: Strengthening the Reporting of Observational Studies in Epidemiology; STRICTA: Standards for Reporting Interventions in Clinical Trials of Acupuncture.

^a^Reference to general editorial policies: whether the journals mentioned to general editorial policies.

^b^Reporting guidance specific in introduction to authors: whether the ‘introduction to authors’ section of a journal provided any reporting guidance.

### Endorsement of reporting guidelines in TM journals

One (*Journal of Korean Acupuncture and Moxibustion Medicine Society*) of the 36 journals (1/36, 2.8%) referred to reporting guidelines in its instruction to authors (Table 1). The website of this journal provided the CONSORT guideline for reporting RCTs as well as the Systematic Reviews of Diagnosis Research (STARD) guideline, the STROBE guideline for observational studies, the Quality of Reporting of Meta-analyses (QUOROM) guideline, the Meta-analyses of Observational Studies (MOOSE) guideline and the Clinical Trial of Acupuncture Intervention (STRICTA) guideline from 2013.

### Assessment of the reporting quality of the included RCTs

#### CONSORT 2010 with non-pharmacological trials

A total of 28 RCT reports were collected from the *Journal of Korean Acupuncture & Moxibustion Medicine Society*, which endorsed a reporting guideline. The majority of the 28 RCTs appraised using the 38-item guideline demonstrated a very low reporting quality because they addressed an average of 11.3 CONSORT items (29.6%). Among the 28 included articles, the reporting percentage in each of the articles was 21.6 to 56.8% (Table 2).

**Table 2:** **The reporting number and percentage for each item according to intervention in endorsing journal for reporting guidelines**

		CONSORT-NPT		STRICTA (Total number of items = 17)	
Intervention	Author (year)	Nunber of reported items	Percent (%)	Number of reported items	Percent (%)
Acupuncture (Total number of items = 37)	Park (2010) [21]	11	29.7	12	70.6
	Lee (2010) [22]	11	29.7	11	64.7
	Kim (2010) [23]	9	24.3	10	58.8
	Kwon (2010) [24]	8	21.6	11	64.7
	Joung (2010) [25]	10	27.0	9	52.9
	Yoon (2010) [26]	9	24.3	14	82.4
	Kim (2010) [27]	10	27.0	17	100.0
	Chung (2010) [28] ^a b^	21	56.8	9	52.9
	Choi (2010) [29]	10	27.0	10	58.8
	Jang (2010) [30]	12	32.4	9	52.9
	Kim (2011) [31] ^b^	13	35.1	9	52.9
	Park (2011) [32]	12	32.4	10	58.8
	Park (2011) [33] ^a^	11	29.7	9	52.9
	Lee (2011) [34]	9	24.3	11	64.7
	Jeong (2011) [35]	9	24.3	9	52.9
	Shin (2011) [36]	10	27.0	7	41.2
	Lee (2011) [37]	12	32.4	9	52.9
	Im (2011) [38]	10	27.0	9	52.9
	Kim (2011) [39]	10	27.0	11	64.7
	Han (2011) [40]	11	29.7	13	76.5
	Kim (2012) [41] ^a^	19	51.4	10	58.5
	Kim (2012) [42]	11	29.7	9	52.9
	Kim (2013) [43]	11	29.7	10	58.8
	Kim (2013) [44] ^a^	19	51.4	17	100.0
Moxibustion (Total number of items = 38)	Cho (2010) [45]	8	21.1		
	Lee (2012) [46]	9	23.7	-	-
	Kim (2013) [47] ^b^	12	31.6	-	-
Cupping (Total number of items = 38)					
	Kim (2013) [48]	9	23.7	-	-
Herbal medicine (Total number of items = 22)					
	Song (2012) [49]	17	77.2	-	-

CONSORT-NPT: Consolidated Standards of Reporting Trials with the non-pharmacological trials extension; STRICTA: Standards for Reporting Interventions in Clinical Trials of Acupuncture.

Values are presented as number of reported items divided by the total number of items.

Intervention on CONSORT-NPT included acupuncture, moxibustion and cupping intervention article besides herbal medicine intervention.

^a^This article included flow participant diagram.

^b^This article included trial registration or protocol information.

Of the 28 included articles, 3 (10.7%) mentioned ‘randomization’ in the title, and 2 (7.1%) described the experimental treatment, comparator, care providers, centers and blinding status. All of the articles (100%) described the scientific background and objective. More than 50% of the articles reported the participants (4a, 4b), the outcomes (6a) in the method section, the participant flow (13a), the implementation of the intervention (a new item in the CONSORT-NPT), the outcomes and estimations (17a) in the results section and the limitations (20) in the discussion section. The other items were assessed in less than 50% of the RCTs. Four items (7a, 7b, 12a and 12b) were not mentioned. The mean percentages for each article section were as follows: the title and abstract section, 8.93%; the introduction section, 100%; the method section, 29.31%; the results section, 40.26%; and the discussion section, 28.57%. The mean percentage for other information was 15.48% and for randomization, 9.82% (Table 3).

**Table 3:** **The reporting number and percentage for each item of CONSORT 2010 checklist with the non-pharmacological trials extension**

Section/topic	Descriptor summary (Additional items from the non-pharmacological trials extension)	28 RCTs included in CONSORT endorsing journal	
		Number of RCTs	Percent (%)
Title and abstract			
	1.a) Identification as a randomized trial in the title	3	10.71
	1.b) Structured summary of trial design, methods, results, and conclusions; for specific guidance (In the abstract, description of the experimental treatment, comparator, care providers, centers and blinding status.)	2	7.14
Total section (average)		2.5	8.93
Introduction			
Background and objectives	2.a) Scientific background and explanation of rationale	28	100
	2.b) Specific objectives or hypotheses	28	100
Total section (average)		28	100
Methods			
Trial design	3.a) Description of trial design (for example, parallel, factorial) including allocation ratio	4	13.79
	3.b) Important changes to methods after trial commencement with reasons	0	0.00
Participants	4.a) Eligibility criteria for participants (When applicable, eligibility criteria for centers and those performing the interventions.)	23	79.31
	4.b) Settings and locations where the data were collected	23	79.31
Interventions	5) Precise details of both the experimental treatment and comparator	-	-
Outcomes	6.a) Completely defined pre-specified primary and secondary outcome measures, including how and when they were assessed	17	58.62
	6.b) Any changes to trial outcomes after the trial commenced with reasons	1	3.45
Sample size	7.a) How sample size was determined (When applicable, details of whether and how the clustering by care providers or centers was addressed.)	0	0.00
	7.b) When applicable, explanation of any interim analyses and stopping guidelines	0	0.00
Total section (average)		8.5	29.31
Randomization			
Sequence generation	8.a) Method used to generate the random allocation sequence (When applicable, how care providers were allocated to each trial group.)	8	28.57
	8.b) Type of randomization; details of any restriction (for example, blocking and block size.)	3	10.71
Allocation concealment	9) Mechanism used to implement the random allocation sequence (for example, sequentially numbered containers), describing any steps taken to conceal the sequence until interventions were assigned	1	3.57
Implementation	10) Who generated the random allocation sequence, who enrolled participants, and who assigned participants to interventions	5	17.86
Blinding	11.a) If done, who was blinded after assignment to interventions (for example, participants, care providers, those assessing outcomes) and how (Whether or not those administering co-interventions were blinded to group assignment. If blinded, method of blinding and description of the similarity of interventions.)	4	14.29
	11.b) If relevant, description of the similarity of interventions	1	3.57
Statistical methods	12.a) Statistical methods used to compare groups for primary and secondary outcomes (When applicable, details of whether and how the clustering by care providers or centers was addressed.)	0	0.00
	12.b) Methods for additional analyses, such as subgroup analyses and adjusted analyses	0	0.00
Total section (average)		2.75	9.82
Results			
Participant flow (A diagram is strongly recommended)	13.a) For each group, the numbers of participants who were randomly assigned, received intended treatment, and were analyzed for the primary outcome (The number of care providers or centers performing the intervention in each group and the number of patients treated by each care provider or in each center.)	23	82.14
	13.b) For each group, losses and exclusions after randomization, together with reasons	5	17.86
Implementation of intervention	Details of the experimental treatment and comparator as they were implemented	25	89.29
Recruitment	14.a) Dates defining the periods of recruitment and follow-up	22	78.57
	14.b) Why the trial ended or was stopped	1	3.57
Baseline data	15) A table showing baseline demographic and clinical characteristics for each group (When applicable, descriptions of care providers (case volume, qualification, expertise, and so on) and centers (volume) in each group.)	10	35.71
Numbers analyzed	16) For each group, number of participants (denominator) included in each analysis and whether the analysis was by original assigned groups	3	10.71
Outcomes and estimation	17.a) For each primary and secondary outcome, results for each group, and the estimated effect size and its precision (for example, 95% confidence interval)	27	96.43
	17.b) For binary outcomes, presentation of both absolute and relative effect sizes is recommended	1	3.57
Ancillary analyses	18) Results of any other analyses performed, including subgroup analyses and adjusted analyses, distinguishing pre-specified from exploratory	1	3.57
Harms	19) All important harms or unintended effects in each group; for specific guidance see CONSORT for harms	6	21.43
Total section (average)		11.3	40.26
Discussion			
Limitations	20) Trial limitations, addressing sources of potential bias, imprecision, and, if relevant, multiplicity of analyses	20	71.43
Generalizability	21) Generalizability (external validity) of the trial findings according to the intervention, comparators, patients and care providers and centers involved in the trial	2	7.14
Interpretation	22) Interpretation consistent with results, balancing benefits and harms, and considering other relevant evidence (In addition, take into account the choice of the comparator, lack of or partial blinding, unequal expertise of care providers or centers in each group.)	2	7.14
Total section (average)		8	28.57
Other information			
Registration	23) Registration number and name of trial registry	2	7.14
Protocol	24) Where the full trial protocol can be accessed, if available	2	7.14
Funding	25) Sources of funding and other support (for example, supply of drugs); role of funders	9	32.14
Total section (average)		4.3	15.48
Total		11.28	29.6

CONSORT-NPT: Consolidated Standards of Reporting Trials with the non-pharmacological trials extension.

Values are presented as number of reported RCTs divided by the total number of eligible RCTs.

Intervention on non-pharmacological trials included moxibustion and cupping intervention article besides acupuncture and herbal medicine intervention.

Of these RCT reports, 4 trials (14.3%) contained flow diagrams, and 3 (10.7%) provided information regarding the trial registration and protocol.

#### STRICTA 2010 for acupuncture interventions trials

The majority of the 24 RCTs (24/29, 82.8%) appraised using the 17 items scored more than 50% in reporting quality by addressing an average of 10.6 STRICTA items (62.5%). Using the STRICTA guidelines, the reporting percentage for each of the articles was 41.18 to 100%, and 2 articles (8.3%) reported all of the items (Table 2).

Among the 24 articles assessed using the 17 items on the STRICTA 2010 guideline, 23 (95.8%) reported the style of acupuncture and the name of the point used and provided a precise description of the control or comparator. A total of 87.5% described the needle type, 79.2% reported the reason for treatment and the needle retention time, 62.5% reported the depth of insertion, 58.3% reported a description of the participating acupuncturists, and 54.2% reported the number of treatment sessions.

The remainder of the items was reported in less than 50% of the RCTs. The mean reporting percentage was 62.5% for the acupuncture rational, 64.3% for the details regarding the reasons for acupuncture being needed, 66.7% for the treatment regimen, and 68.8% for the control or comparator interventions (Table 4).

**Table 4:** **The reporting number and percentage for each item of STRICTA 2010 checklist of information**

Items	Detail	24 acupuncture trials	
		Number of RCTs	Percent (%)
1. Acupuncture rationale	1a) Style of acupuncture	23	95.8
	1b) Reasoning for treatment provided, based on historical context, literature sources, and/or consensus methods, with references where appropriate	19	79.2
	1c) Extent to which treatment was varied	3	12.5
Total section (average)		15	62.5
2. Details of needling	2a) Number of needle insertions per subject per session (mean and range where relevant)	11	45.8
	2b) Names (or location if no standard name) of points used (uni/bilateral)	23	95.8
	2c) Depth of insertion, based on a specified unit of measurement, or on a particular tissue level	15	62.5
	2d) Response sought	4	16.7
	2e) Needle stimulation	15	62.5
	2f) Needle retention time	19	79.2
	2g) Needle type	21	87.5
Total section (average)		15.4	64.3
3. Treatment regimen	3a) Number of treatment sessions	13	54.2
	3b) Frequency and duration of treatment sessions	19	79.2
Total section (average)		16	66.7
4. Other components of treatment	4a) Details of other interventions administered to the acupuncture group (for example, moxibustion, cupping, herbs, exercises, lifestyle advice)	17	70.8
	4b) Setting and context of treatment, including instructions to practitioners, and information and explanations to patients	6	25.0
Total section (average)		11.5	47.9
5. Practitioner background	5) Description of participating acupuncturists (qualification or professional affiliation, years in acupuncture practice, other relevant experience)	14	58.3
Total section (average)		14	58.3
6. Control or comparator interventions	6a) Rationale for the control or comparator in the context of the research question, with sources that justify this choice	10	41.7
	6b) Precise description of the control or comparator. If sham acupuncture or any other type of acupuncture-like control is used, provide details as for items 1 to 3 above.	23	95.8
Total section (average)		16.5	68.8
Total		10.6	62.5

STRICTA: Standards for Reporting Interventions in Clinical Trials of Acupuncture.

This checklist is designed to replace CONSORT 2010’s item 5 when reporting an acupuncture trial.

Values are presented as number of reported RCTs divided by the total number of eligible RCTs.

#### The CONSORT extension for herbal intervention trials

One RCT was assessed using the recommendations for the reporting of herbal interventions. Of the 22 items, 17 items (77.27%) were reported. Of the 6 intervention items, the following 4 items (66.7%) were reported: the product name, dosage regimen and quantitative description, placebo/control group and practitioner.

#### Comparison after journal adoption of reporting guidelines

Table 5 compares the reporting of the guideline items in the RCTs from the journals that did or did not endorse the CONSORT and STRICTA guidelines. The percentages of the reported items in the RCTs (the percentage difference, 21.1; 95% CI, 6.6 to 35.5) were better after the adoption of the STRICTA reporting guidelines, with statistical significance (*P* = 0.0042). In the CONSORT-promoting journals, the completeness of the reporting increased slightly (percentage difference, 3.1%; 95% CI, -5.0 to 11.2) after endorsement of the guidelines by the journal, without a statistically significant difference (*P* = 0.4592) (Table 5).

**Table 5:** **Comparison of reported items in journals that did and did not promote adherence to CONSORT and STRICTA statement**

	CONSORT-NPT		STRICTA	
	Before 2013	After 2013	Before 2013	After 2013
Number of items reported/total number of items	282/912	51/150	228/391	27/34
Percent reported items in RCTs (n/N)^a^	30.9	34.0	58.3	79.4
Difference in percent between the two groups (CI)^b^	3.1 (-5.0 to 11.2)		21.1 (6.6 to 35.5)	
*P*-value	0.4592		0.0042	

CONSORT-NPT: Consolidated Standards of Reporting Trials with the non-pharmacological trials extension; STRICTA: Standards for Reporting Interventions in Clinical Trials of Acupuncture; RCT: randomized controlled trials.

^a^n/N: number of times reported/total number of items evaluated.

^b^95% confidence intervals (%).

*P*-value calculated for each of these before after comparisons.

## Discussion

This study is the most comprehensive review of the reporting guidelines in Korean TM journals. We utilized a highly sensitive approach to the endorsement of reporting guidelines by Korean TM journals and conducted a complete assessment of the reporting quality of the trials published in journals that endorsed reporting guidelines. In this study, 36 core journals were selected after investigating the endorsement of reporting guidelines and the uniform requirements for manuscripts (URM) of these Korean TM journals. Our study has shown that most Korean TM journals provide little or no guidance regarding the information to report in describing research dependent on study design and interventions. One journal (the *Journal of Acupuncture and Moxibustion Society*) has referenced the reporting guidelines in its instructions for authors since 2013. Additionally, we found that the majority of the articles failed to follow the reporting guidelines. Fifteen journals mentioned the ICMJE or the URM.

An important aspect of RCT quality is related to the consideration of randomization; however, in our study, all of the items are in very low percentages (9.82%). We hypothesized that treatment in TM is a unique and complex intervention, and difficulties in blinding and allocation concealment are significantly influenced by the care providers’ expertise and the care center’s volume of treatment. A total of 4.3% of the studies referenced the trial registration number, protocol and funding source, which indicates journal editors have not focused on these items.

The *Journal of Korean Acupuncture and Moxibustion Medicine Society* is a core journal that is highly cited in Korea. In this journal, we found that 29 parallel RCTs of the 105 assessed RCTs did not report the number of care providers and centers in each group. For non-pharmacological treatments, the number of care providers and centers in each group and the number of patients treated by each care provider is essential so that the biological and statistical significance of the results could be assessed or the data reanalyzed. ‘Implementation of intervention’ is a new item added to the CONSORT-NPT 2010 statement. Because participants and care providers are frequently not blinded to treatment assignment in NPT, a risk for unequal administration of additional treatments and contamination might influence the estimates of the treatment effect [9]. In our study, 25 RCTs (89.3%) provided details regarding the manner in which the intervention was actually administered.

The CONSORT-NPT statement has not been translated into Korean; however, the STRICTA guidelines have been translated into Korean and were published in our endorsing journal in 2001 [50]. In addition, the translation could be found on the revised STRICTA website [51]. It is assumed that Korean journal editors and authors have simple access to the STRICTA items when reporting on acupuncture trials. Our findings showed that the STRICTA items (10.6/17, 62.5%) are more completely reported than the CONSORT-NPT items (11.28/37, 29.6%).

To adequately translate research into practice, research results should be reported by a method that is useful to practicing clinicians and policymakers. Based on evidence from systematic reviews, the implementation of reporting guidelines such as CONSORT for randomized controlled trials might improve the quality of research reporting. Other reporting guidelines similar to the CONSORT statement provide advice on reporting research methods and findings for other types of study designs [52].

Several studies [53–55] have assessed the effect of using the CONSORT statement to improve the reporting of RCTs. These studies suggested that journal endorsement of CONSORT might improve the completeness of reporting in the RCTs they publish. In our study, we found that RCTs published after reporting guidelines were adopted by journals were more likely to include the required reporting items shown in Table 5. Despite the relative improvements when CONSORT is endorsed by journals, the completeness of reporting remains suboptimal. In particular, Korean TM journals should guide their authors towards reporting guidelines, which have the potential to improve the quality of reporting and consequently the quality of research.

This study identifies several specific tasks required to improve the quality of clinical trials. First, all Korean TM Journals should be more vigilant regarding the information in their instructions to authors and explicit in their expectations of adherence to specific recommendations; additionally, they should cite the web address for the guidelines they follow to ensure that the latest versions and extensions are obtained. Second, in the initial submission stage, TM authors should adhere to the reporting guidance for the study design and intervention. Third, several reporting guidelines exist, and TM practitioners and researchers should consider the other interventions and develop the formal endorsement of guidelines for the TM intervention. Fourth, improved education and awareness among all of the stakeholders and hard-wired compliance through electronic journal submission systems could benefit the quality of clinical trials.

This study has the following limitations. First, because we found that only one Korean TM journal has adopted reporting guidelines, we retrieved only 29 RCTs for assessment, and the results could not fully represent all of the Korean TM journals. We could hypothesize that journals, RCTs or study designs not mentioned in this study have little potential for better reporting. This hypothesis is likely because many reporting guidelines have not been recommended by Korean TM journals. Second, our measures of study quality depend on the information reported in an article, and no attempt was made to judge the clinical merits or assumption models in the analyses. Third, no telephone calls were made and no Emails were sent to the editorial offices of the journals investigated; therefore, we do not know the opinions of the journal editors regarding the issues addressed in this study.

Our data suggest the need for TM journals to adopt reporting guidelines because better reporting is likely to influence the quality as well as the effect of future research. Korean researchers could provide robust evidence to establish health care standards for clinical practice. TM researchers in Korea should exert significant effort in improving the number and quality of primary studies by considering the study design and unique treatment intervention system in reference to reporting guidelines.

## Conclusions

The endorsement of reporting guidelines is limited in TM journals in Korea, and many items in research studies were far from satisfactory. We hope that all of the TM journals will support reporting guidelines by registering on the websites of the organizations that have established reporting guideline statements. This article should generate further research regarding the mechanism for improving the quality of RCT reporting. Interested readers, reviewers, researchers, and editors in Korea could use the reporting guide statements and generate theories for improving research.

## Abbreviations

AEC: Animal Ethical Committee
CAM: complementary and alternative medicine
CONSORT-NPT: Consolidated Standards of Reporting Trials - statement for non-pharmacological trials
COPE: Committee on Publication Ethics
ICMJE: International Committee of Medical Journal
MOOSE: Meta-analyses of Observational Studies
PRISMA: Preferred Reporting Items for Systematic Reviews and Meta-Analyses
QUOROM: Quality of Reporting of Meta-analyses
RCTs: randomized controlled trials
STARD: Systematic Reviews of Diagnosis Research
STRICTA: Standards for Reporting Interventions in Clinical Trials of Acupuncture
STROBE: Strengthening the Reporting of Observational Studies in Epidemiology
TM: traditional medicine
URM: Uniform Requirements for Manuscripts.
